# First person – Eleni Christoforidou

**DOI:** 10.1242/dmm.050873

**Published:** 2024-05-29

## Abstract

First Person is a series of interviews with the first authors of a selection of papers published in Disease Models & Mechanisms, helping researchers promote themselves alongside their papers. Eleni Christoforidou is first author on ‘ An ALS-associated mutation dysregulates microglia-derived extracellular microRNAs in a sex-specific manner’, published in DMM. Eleni is a Research Fellow in the lab of Prof. Majid Hafezparast at Sussex Neuroscience, School of Life Sciences, University of Sussex, Brighton, UK, and is interested in identifying biomarkers for the prognosis and progression of amyotrophic lateral sclerosis (ALS).


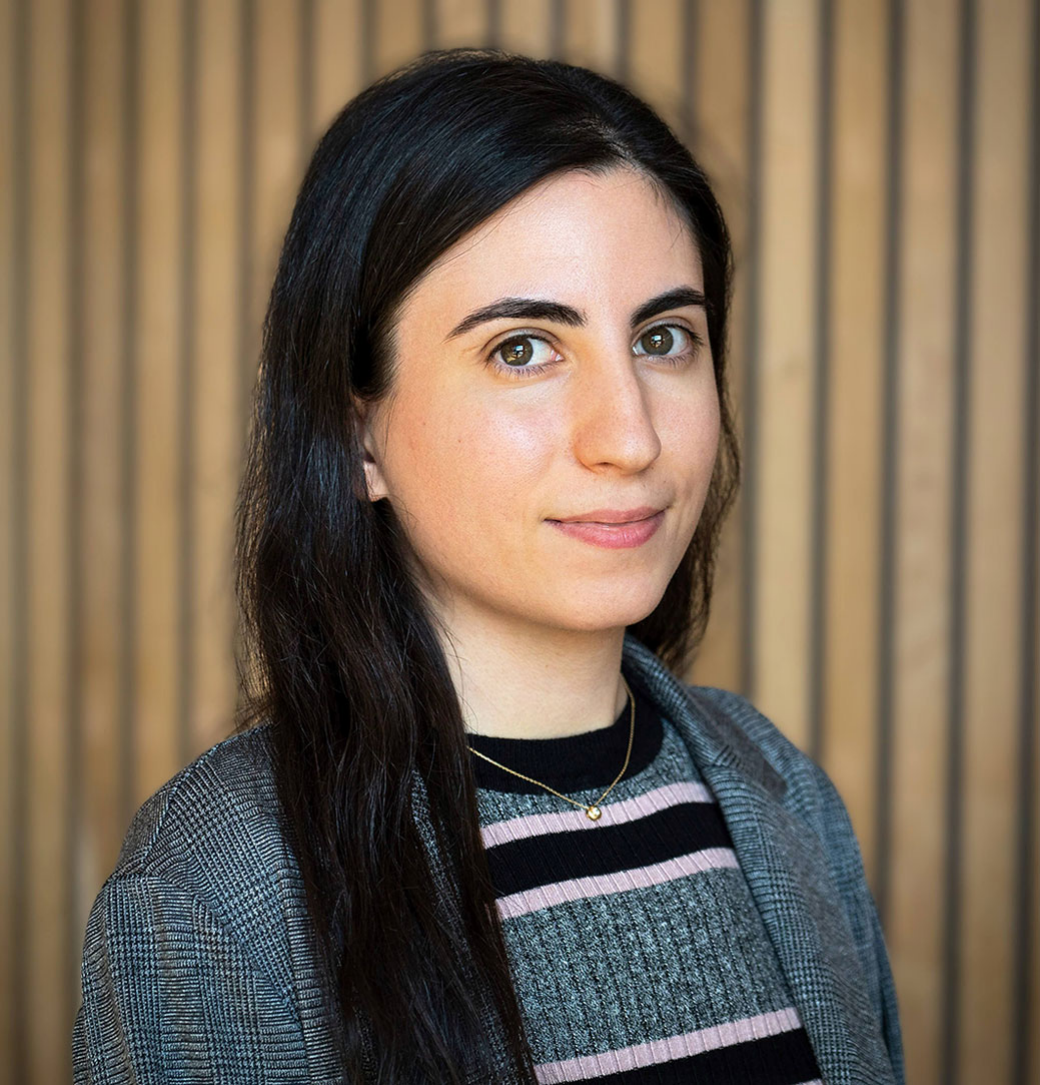


**Eleni Christoforidou**

**Who or what inspired you to become a scientist?**

From a young age, the complexity and mysteries of living organisms, particularly the human brain, deeply intrigued me and sparked my decision to become a scientist. I was particularly captivated by the way mental processes could be influenced by neurological conditions, an interest sparked by volunteer work at a local child development centre during my teenage years. Observing doctors and researchers work tirelessly to diagnose and treat neurological disorders reinforced my desire to join their ranks. This inspired me to pursue a degree in neuroscience.

**What is the main question or challenge in disease biology you are addressing in this paper? How did you go about investigating your question or challenge?**

Our study focuses on how a TDP-43 mutation linked to amyotrophic lateral sclerosis (ALS) alters microRNA release from microglia, potentially contributing to the pathogenesis of the disease. We particularly examined the impact of the mutation under inflammatory conditions induced by a pro-inflammatory stimulus, and observed how these effects vary between males and females. By utilising next-generation sequencing to profile microRNA expression, we identified key differences in microRNA profiles that are influenced by the mutation in a sex-dependent manner. Our results suggest that the mutated gene significantly affects microRNA release from microglia, which could be crucial for understanding and managing the progression of ALS.

**How would you explain the main findings of your paper to non-scientific family and friends?**

In our study, we looked at a specific gene mutation that is linked to a serious nerve disease called ALS, which affects muscle control and leads to paralysis. We wanted to understand how this mutation might cause problems in the immune cells of the brain, which help protect and support nerve functions. We found that when these immune cells have the mutation, they behave differently, and this difference is more noticeable in females than in males. We discovered that these immune cells release tiny molecules that could influence other cells, and these molecules are released differently when the mutation is present. When these molecules are released in abnormal amounts because of the mutation, it could contribute to the disease. This is important because it helps us understand more about how ALS might progress and opens new possibilities for targeting these changes to develop treatments.

Our study demonstrates how a mutation linked to ALS causes specific changes in microglia […] By altering the release of microRNAs in a sex-specific manner, the mutation could contribute to disease progression differently in men and women.

**What are the potential implications of these results for disease biology and the possible impact on patients?**

Our study demonstrates how a mutation linked to ALS causes specific changes in microglia, the immune cells of the brain, which could advance our understanding of the disease. By altering the release of microRNAs in a sex-specific manner, the mutation could contribute to disease progression differently in men and women. This finding is crucial because it suggests that treatments could be tailored based on an individual's genetic profile and sex, potentially improving outcomes for patients with ALS by targeting these specific molecular changes.

**Figure:**
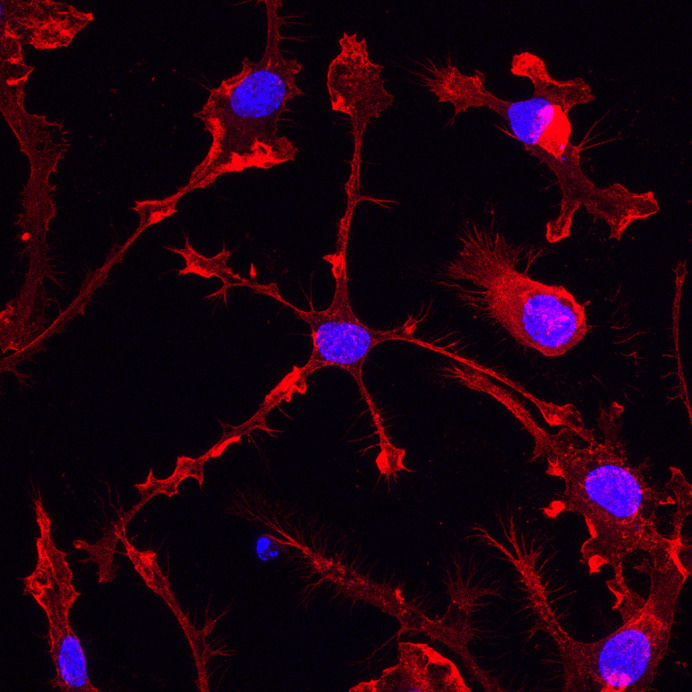
Brain microglia grown in a dish, with nuclei shown in blue.

**Why did you choose DMM for your paper?**

We chose DMM for our paper because the journal has a strong focus on publishing detailed studies that explore the mechanisms of diseases through innovative models. Our study, which investigates the specific genetic and molecular mechanisms contributing to ALS, aligns perfectly with the journal's commitment to advancing the understanding of disease processes. Additionally, the journal's interdisciplinary audience ensures that our findings reach researchers and clinicians across various fields, potentially fostering collaborative efforts to develop targeted therapies based on our research.

**Given your current role, what challenges do you face and what changes could improve the professional lives of other scientists in this role?**

One of the main challenges in my role is securing stable funding to support long-term research projects. This uncertainty can be stressful and may deter talented researchers from pursuing a career in academic research. To improve the professional lives of scientists in similar roles, institutions could provide more robust funding models and grant programs that ensure continuity and stability. Additionally, enhancing collaborative networks with industry partners could open alternative funding sources and create more opportunities for translational research.

**What's next for you?**

Moving forward, I am excited to delve into a new aspect of motor neuron disease research by identifying potential biomarkers that could help predict the progression of ALS in patients. This project involves analysing non-coding RNAs, such as microRNAs, from human samples collected in a clinical trial. The goal is to pinpoint specific biomarkers that could not only improve our understanding of ALS but also potentially lead to better diagnostic tools and personalized treatment plans for patients. This work builds directly on my previous research, applying it in a clinical context to make a tangible difference in the management of the disease.

**Tell us something interesting about yourself that wouldn't be on your CV**

An interesting detail about me that you wouldn't find on my CV is that I speak six languages, having learned most of them throughout my schooling. This has enriched my ability to communicate with a diverse group of people and understand different cultural perspectives, which has been invaluable in my research career.
